# Iatrogenic diversion of the inferior vena cava into the left atrium diagnosed 43 years after surgical atrial septal defect closure

**DOI:** 10.1002/ccr3.4618

**Published:** 2021-08-16

**Authors:** Toshio Doi, Kanetsugu Nagao, Akihiko Higashida, Masaya Aoki, Shigeki Yokoyama, Shigeyuki Yamashita, Akio Yamashita, Kazuaki Fukahara, Naoki Yoshimura

**Affiliations:** ^1^ First Department of Surgery University of Toyama Toyama Japan

**Keywords:** cyanosis, diversion of the inferior vena cava into the left atrium, right‐to‐left shunt, surgical closure of atrial septal defect

## Abstract

The diversion of the inferior vena cava into the left atrium after surgical atrial septal defect closure is a fatal complication. Cases of atrial septal defect with no inferior rim should be treated with this complication in mind.

## Graphical abstract

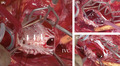

## 1 INTRODUCTION

The diversion of the inferior vena cava into the left atrium after surgical atrial septal defect closure is a fatal complication. Cases of atrial septal defect with no inferior rim require the management of experienced surgeons and should be treated with this complication in mind.

Surgical closure of atrial septal defects (ASDs) has been reported to be safe and is associated with a low incidence of fatal complications.^1^, ^2^, ^3^ However, in cases such as low‐lying large ASD with no inferior rim, the rare and serious complication of iatrogenic diversion of the inferior vena cava (IVC) into the left atrium (LA) has been reported.^4^, ^5^, ^6^, ^7^, ^8^, ^9^, ^10^, ^11^, ^12^ Herein, we report the case of an adult woman who underwent surgical closure of ASD in childhood and was reoperated on 43 years later for diversion of the IVC into the LA.

## 2 CASE REPORT

A 47‐year‐old woman visited her local physician with chief complaints of shortness of breath and cyanosis during exercise, which she had been aware of for several years. She had a history of surgical ASD closure at the age of 4 years at another hospital. Transthoracic echocardiographic examination by her local physician revealed two residual ASDs, and she was referred to our hospital for further examination and treatment, including reoperation. Her parents informed us that her postoperative respiratory status had become unstable, requiring tracheostomy and long‐term ventilator management. We contacted the hospital where the surgery was performed to inquire about the surgery and postoperative course, but no records were kept, and the details were unknown. She had been followed up as an outpatient for several years after the surgery but had not visited a medical institution since then. Thereafter, she avoided strenuous exercise and went about her daily life but gradually developed shortness of breath and cyanosis during exercise. She had been married but had not become pregnant or gave birth.

**FIGURE 1:**
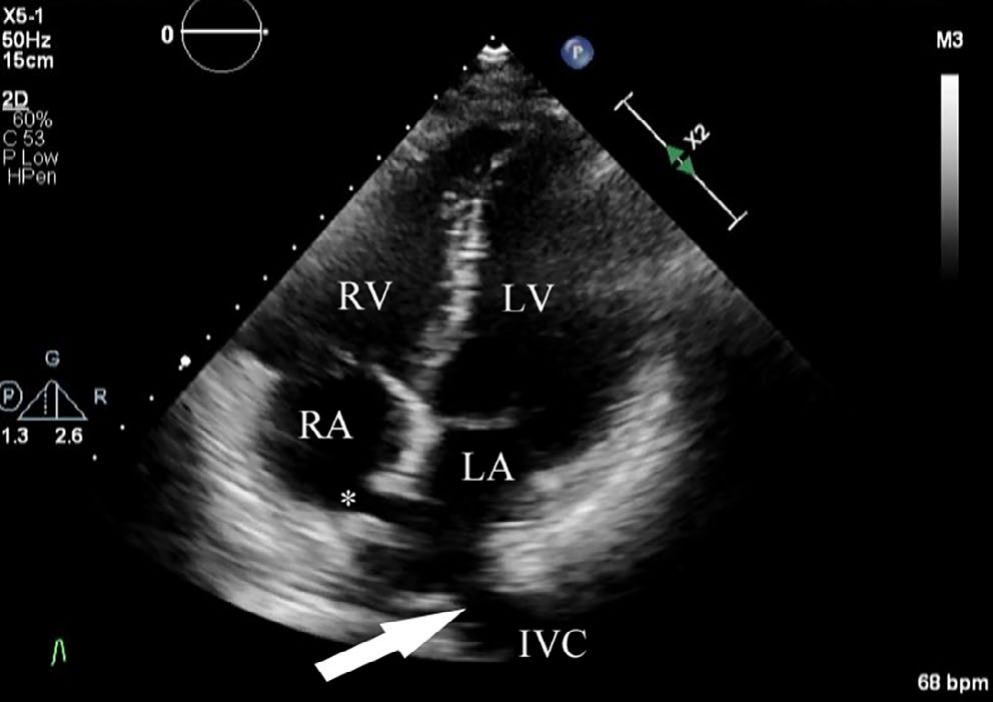
Preoperative transthoracic echocardiography image: The apical 4‐chamber view demonstrates residual atrial septal defects (asterisk) with the inferior vena cava directed to the left atrium (arrow). IVC, inferior vena cava; LA, left atrium; LV, left ventricle; RA, right atrium; RV, right ventricle

**FIGURE 2:**
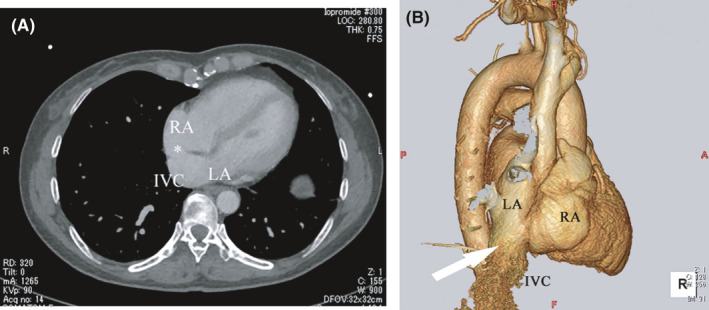
Preoperative contrast‐enhanced computed tomography images. (A) The axial view displays residual atrial septal defects (asterisk) with the inferior vena cava being directed to the left atrium (arrow). (B) The three‐dimensional view demonstrates a direct connection of the inferior vena cava and the left atrium (arrow). IVC, inferior vena cava; LA, left atrium; RA, right atrium

At her initial visit to our hospital, her blood pressure and pulse rate were normal. She had no cyanosis at rest but had lip cyanosis and complained of mild respiratory distress during exercise. Her resting SpO_2_ was 92% on room air and decreased to 77% during exercise. There were no other abnormalities on physical examination. Blood tests revealed a hemoglobin of 14.3 g/dl and hematocrit of 41.5%. Transthoracic echocardiography demonstrated two residual ASDs (8.9 mm and 11.2 mm) with a left‐to‐right shunt, and the IVC connected to the LA, not the right atrium (RA; Figure 1). Contrast‐enhanced computed tomography (CT), including three‐dimensional CT (3D‐CT), clearly demonstrated that the IVC was connected to the LA (Figure 2). The patient was diagnosed with an iatrogenic diversion of the IVC into the LA in addition to residual ASD after previous surgical closure. Therefore, revision surgery was performed.

**FIGURE 3:**
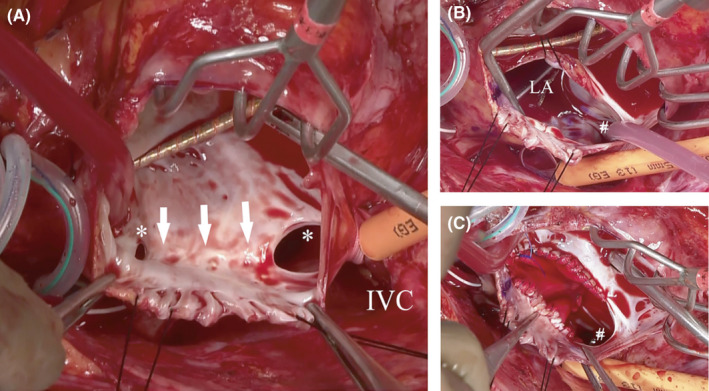
Intraoperative findings demonstrating (A) two residual atrial septal defects (asterisk) and the direct suture line (arrow) previously used to close the atrial septal defect. (B) The orifice of the inferior vena cava (hash) is visualized in the left atrium after opening the atrial septum. (C) Plasty of the inferior rim of the atrial septal defect and atrial septoplasty using a Dacron patch to displace the inferior vena cava (hash) to the right atrial side. IVC, inferior vena cava; LA, left atrium

Surgery was performed through a full median re‐sternotomy. After systemic heparinization, extracorporeal cardiopulmonary circulation (ECC) was established with an arterial cannula in the ascending aorta and venous drainage from the direct superior vena cava (SVC) and femoral vein (FV). After the ascending aorta was clamped, cardiac arrest was achieved by antegrade infusion of a cooled blood cardioplegic solution. The SVC and IVC were snared, and a right atrial incision was made. The suture line performed at the time of the previous surgery was found in the atrial septum, suggesting that the ASD was directly sutured without patching (Figure 3A). A large 5‐mm residual ASD was found at the superior edge of the suture line, and a 15‐mm residual ASD was found at the inferior edge (Figure 3A). A large longitudinal incision was made in the atrial septum between the two residual ASDs, and we were able to identify the orifice of the IVC in the LA (Figure 3B). We performed plasty of the inferior rim of the ASD and atrial septoplasty using a Dacron patch to reroute the orifice of the IVC to the right atrial side (Figure 3C). The patient was successfully weaned from the ECC, and intraoperative transesophageal echocardiography confirmed no evidence of residual ASD with all blood flow from the IVC returning to the RA. The operative time, ECC time, and cardiac arrest time were 232, 118, and 70 min, respectively. Her postoperative course was favorable, and cyanosis or decrease in SpO_2_ was no longer observed during exercise. The patient was discharged unaided on postoperative day 18.

## 3 DISCUSSION

Surgical ASD closure is a well‐established and safe procedure. Minor complications such as supraventricular arrhythmias—including atrial fibrillation and atrial flutter, pericardial effusion, and a small amount of residual shunting—have been reported, but postoperative death or serious complications are rare.^1^, ^2^, ^3^ There are only a few case reports describing IVC diversion into the LA as a complication, and its actual frequency is unknown.^4^, ^5^, ^6^, ^7^, ^8^, ^9^, ^10^, ^11^, ^12^, ^13^, ^14^In previous reports, this complication was suspected based on the appearance of postoperative cyanosis, and in many cases, the diagnosis was confirmed by contrast echocardiography and IVC angiography from the FV.^4^, ^5^, ^6^, ^7^, ^8^, ^9^, ^10^, ^11^ However, the accuracy of cardiac magnetic resonance imaging (MRI) and enhanced 3D‐CT has improved in recent years, and these noninvasive modalities have been reported to be useful in diagnosing this complication.^11^, ^12^, ^13^ In this case, the complication was suspected on transthoracic echocardiography after the appearance of cyanosis, and the diagnosis was confirmed by 3D‐CT. This rare complication can be diagnosed immediately after surgery^4^, ^10^, ^14^ or may only be noticed after many years.^5^, ^6^, ^7^, ^8^, ^9^, ^11^, ^12^, ^13^ Factors that may delay diagnosis until the remote phase include partial diversion, development of collateral blood flow via the azygos vein with stenosis of the IVC, and the presence of a left‐to‐right shunt through the residual ASD.^9^, ^11^, ^12^ In this case, partial disruption of the ASD suture line at some point postoperatively and the development of a residual left‐to‐right shunt, which allowed part of the IVC blood flow to return to the RA, were thought to be the reason for the delay in detection.

Lower‐lying large ASDs with no inferior rim, inferior sinus venosus type ASD, and inferior ASD complicated by partial anomalous pulmonary venous return are the types that are most prone to diversion of the IVC.^4^, ^5^, ^6^, ^7^, ^8^ Especially in patients with a developed eustachian valve, this complication is caused by misidentification of the valve as the inferior rim of the ASD and its inclusion in the suture line for ASD closure.^4^, ^5^, ^6^, ^7^, ^8^When diversion of the IVC into the LA occurs, patients become cyanotic due to a severe right‐to‐left shunt. This leads to a marked decrease in exercise tolerance and limitations in daily life. It also carries the risk of paradoxical cerebral embolism.^9^, ^11^ Cerebral embolism may often be fatal and leads to further deterioration in activities of daily living. Moreover, in young female patients, cyanosis can be problematic for pregnancy and childbirth because of its adverse effects on the mother and fetus.^15^ Therefore, surgical intervention is necessary as soon as the diagnosis is made. Usually, re‐surgical repair is repeated under ECC to allow IVC blood flow into the RA. There have been reports of transcatheter treatment, but this may be limited to patients who cannot tolerate open‐heart surgery.^13^Diversion of the IVC is a technical complication that must be avoided. Possible preventive methods include (1) increasing awareness of this complication among surgeons; (2) obtaining a good field of view and observing the anatomy around the ASD carefully; (3) inserting the drainage cannula directly into the IVC or from the FV to secure the view of the inferior rim of the ASD^5^, ^14^; (4) releasing the IVC snare before right atrial closure to ensure that the IVC flow enters the RA^5^, ^11^; (5) and having an experienced anesthesiologist check the IVC flow using intraoperative transesophageal echocardiography.^14^ These measures can help avoid this particular complication, even if they prolong the operative time.

## 4 CONCLUSION

We encountered a case of diversion of the IVC into the LA after surgical closure of an ASD, which is rare but has a severe postoperative course. Surgical closure of ASDs is often performed by young surgeons. In recent years, minimally invasive approaches have also been employed in light of their superior cosmetic outcomes. If the ASD is closed by an inexperienced surgeon using a minimally invasive approach with a poor visual field, the risk of this complication may increase. Therefore, cases of ASD with no inferior rim require treatment by an experienced surgeon while keeping this complication in mind.

## CONFLICT OF INTEREST

None declared.

## AUTHOR CONTRIBUTIONS

TD involved in data collection and manuscript drafting and preparation. KN, AH, MA, S Yokoyama, S Yamashita, AY, and KF analyzed the data and revised the manuscript. NY edited the final manuscript.

## ETHICAL APPROVAL

Informed consent to publish the case details was obtained from the patient.

## Data Availability

The data that support the findings of this study are available from the corresponding author upon reasonable request.
